# Clinical Translationality of *KCNJ5* Mutation in Aldosterone Producing Adenoma

**DOI:** 10.3390/ijms23169042

**Published:** 2022-08-12

**Authors:** Takumi Kitamoto, Tetsuo Nishikawa

**Affiliations:** 1 Endocrinology and Diabetes Center, Yokohama Rosai Hospital, Yokohama 2220036, Japan; 2 Department of Endocrinology, Hematology and Gerontology, Chiba University Graduate School of Medicine, Chiba 2608670, Japan; 3 Nishikawa Clinic, Yokohama 2220033, Japan

**Keywords:** primary aldosteronism, KCNJ5, aldosterone producing adenoma, genetic abnormality

## Abstract

Hypertension due to primary aldosteronism poses a risk of severe cardiovascular complications compared to essential hypertension. The discovery of the *KCNJ5* somatic mutation in aldosteroene producing adenoma (APA) in 2011 and the development of specific CYP11B2 antibodies in 2012 have greatly advanced our understanding of the pathophysiology of primary aldosteronism. In particular, the presence of CYP11B2-positive aldosterone-producing micronodules (APMs) in the adrenal glands of normotensive individuals and the presence of renin-independent aldosterone excess in normotensive subjects demonstrated the continuum of the pathogenesis of PA. Furthermore, among the aldosterone driver mutations which incur excessive aldosterone secretion, *KCNJ5* was a major somatic mutation in APA, while *CACNA1D* is a leading somatic mutation in APMs and idiopathic hyperaldosteronism (IHA), suggesting a distinctive pathogenesis between APA and IHA. Although the functional detail of APMs has not been still uncovered, its impact on the pathogenesis of PA is gradually being revealed. In this review, we summarize the integrated findings regarding APA, APM or diffuse hyperplasia defined by novel CYP11B2, and aldosterone driver mutations. Following this, we discuss the clinical implications of *KCNJ5* mutations to support better cardiovascular outcomes of primary aldosteronism.

## 1. Introduction

**Figure 1:**
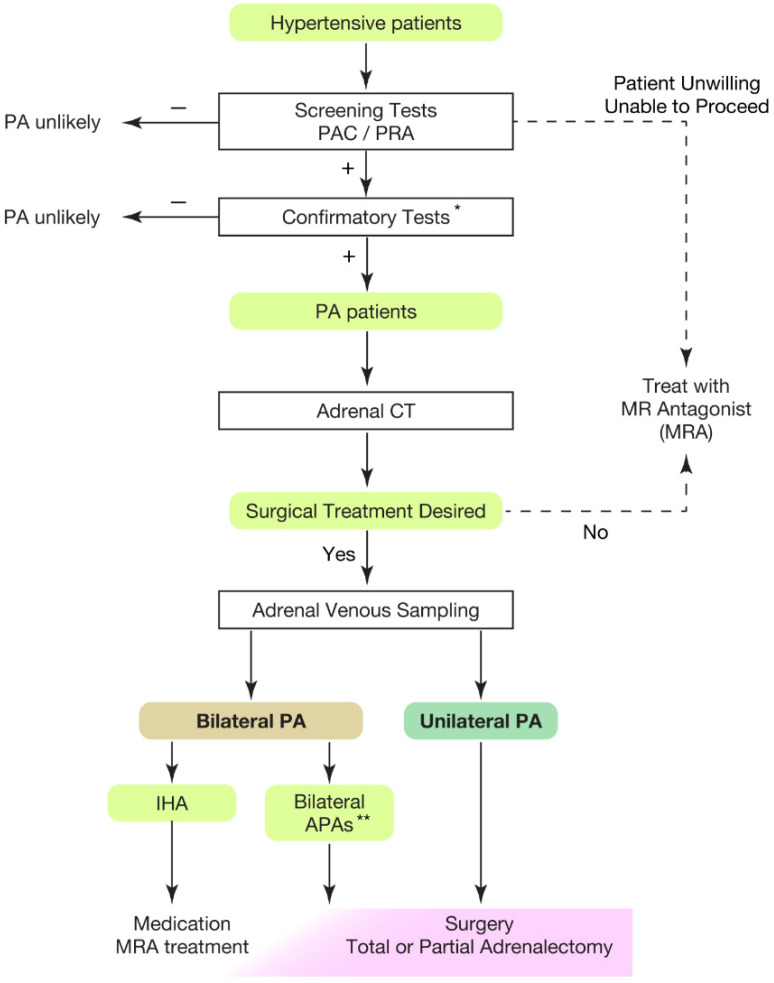
Overview of case detection and treatment strategy of primary aldosteronism. Most guidelines recommend to use plasma aldosterone-renin ratio (PAC/PRA) for screening test [21,22,23,24,33]. * Confirmatory tests included captopril challenge test, saline infusion test, and oral sodium loading test. ** Bilateral APAs can be diagnosed by segment selective adrenal venous sampling [34,35]. Partial adrenalectomy to spare normal adrenal tissue should be considered for the dominant side of APA if attempting surgical treatment. This would be helpful when the residual APA may cause MRA-resistant PA in the future. PAC, plasma aldosterone concentration; PRA, plasma renin activity; PA, primary aldosteronism; MRA, mineral corticoid receptor antagonist; CT, computed tomography; IHA, idiopathic hyperaldosteronism; APA, aldosterone producing adenoma.

Hypertension is the leading risk factor for death and disability in the global population [1,2,3,4]. The treatment goal of hypertensive patients is to maintain a healthy life expectancy comparable to that of healthy individuals. To achieve it, we need to cure the disease and reduce the risk of cardiovascular complications as much as possible. In this regard, an appropriate classification of hypertension is necessary. Secondary hypertension should be screened for the patients showing following characteristics [5,6,7]: (1) onset of hypertension (<30 years), (2) abrupt onset of hypertension, (3) drug-resistant hypertension, (4) exacerbation of previously controlled hypertension, (5) suspicion of endocrine causes of hypertension or CKD, (6) clinical features suggestive of obstructive sleep apnoea, (7) unprovoked or excessive hypokalemia, (8) disproportionate targeted organ damage for degree of hypertension, (9) onset of diastolic hypertension in older adults (≥65 years). Primary aldosteronism (PA) is found in 5–7% of all hypertensive patients [8,9,10,11,12,13] and even explains 20% of patients with third-degree hypertension [14]. Despite its frequency, less than 1% of patients with PA undergo screening and progress to treatment throughout their lifetime [15,16,17,18,19,20]. One reason for the underdiagnosis may be that health care providers are not fully aware of the high frequency of PA in hypertensive patients. In addition, there is not sufficient consensus on the targets and methods of screening tests. In most of the institutions, patients to be screened are those with severe hypertension [21,22,23], i.e., in addition to the characteristics indicating secondary hypertension, (1) hypertension and spontaneous or diuretic-induced hypokalemia, (2) hypertension and adrenal incidentaloma, (3) atrial fibrillation in the absence of structural heart disease, (4) a family history of early onset hypertension or stroke at a young age (<40 years), (5) all hypertensive first-degree relatives of patients with PA. Others recommend screening for all hypertensive patients [24] from a cost-effectiveness perspective based on its high frequency [25,26] and the need for lifelong antihypertensive medication. With suspicions of PA at the first screening test evaluating plasma aldosterone levels and plasma renin activity, we should move to definitive diagnosis of PA, performing confirmatory tests (Figure 1). If the patient desires surgical treatment, subtype diagnosis should be considered, which requires adrenal venous sampling (AVS). However, AVS is available at limited institutions, therefore, efforts have been made to identify cases in which AVS can be bypassed and to determine the indications for surgery by CT scan [27,28,29,30]. It should be noted, however, that the results of CT imaging and AVS differ in 28–38% of cases [31,32]. Thus, AVS has been recommended for precise subtype diagnosis to date. In this review, we summarize integrated findings regarding new insight of pathophysiology of PA and discuss clinical transferability of the findings, particularly focusing on somatic *KCNJ5* mutation. We searched MEDLINE for articles published 1 January 1955 to 1 June 2022, using the search terms “primary aldosteronism”, “Conn’s syndrome”, “hyperaldosteronism”, “adrenal vein sampling”, and “genetics”. We mainly focused on the publications in the past 5 years (1 June 2017, to 1 June 2022) in the English language, and selected relevant and highly referenced publications before this time.

## 2. Cardiovascular Outcome of Primary Aldosteronism

PA leads to abnormalities in the renin-angiotensin-aldosterone system and is known to increase the incidence of atrial fibrillation, heart failure, and stroke by 3.52, 2.05, and 2.58 times compared to hypertension due to essential hypertension [36,37,38,39]. Appropriate treatment for PA can greatly improve the prognosis of cardiovascular complications [38], and thus early diagnosis of PA and specific treatment for the condition are important. The etiology of PA is caused by two broad categories: aldosterone-producing adenomas (APA) and idiopathic hyper aldosteronism (IHA). In the former, the pathogenetic localization is clear, and surgical resection can be expected to cure the disease [40]. The frequency of APA and IHA has been reported to vary between primary care and referral centers [11,12,41], but it is difficult to determine the exact frequency. APA should be pathologically diagnosed by morphology and staining for CYP11B2 [42], an aldosterone-producing rate-limiting enzyme. However, surgical resection is only performed in cases who want to receive surgery and have been pre-surgically diagnosed with APA by adrenal venous sampling (AVS). IHA, on the other hand, is diagnosed by AVS and is usually treated with medication. We, therefore, cannot rule out the possibility of mixed misdiagnosis of APA in most cases. The frequency of APA and IHA diagnoses depends largely on the availability, procedure (e.g., usage of ACTH stimulation) and diagnostic criteria of AVS. Recently, tributary adrenal vein in addition to central adrenal vein sampling (a.k.a., segment selective AVS) has revealed that more than 10% of patients were elucidated as having APA, which could not be diagnosed by classic AVS evaluation of left-right difference [34]. Therefore, it is important to identify the localization of increased aldosterone synthesis within the adrenal gland to accurately diagnose APA, rather than simply looking at the right or left side of the adrenal gland. However, as a practical matter, the number of facilities where AVS is available is still far less than the expected number of patients with PA, and it is difficult to perform AVS on all patients with PA. Furthermore, publications reporting use of segment selective AVS are mostly from Japan [34,35,43,44]. Standardization and cost reduction of the AVS technique would give us precise etiology of APA and IHA. The data would help us to develop the diagnostic algorithm to predict the cases who require AVS.

## 3. Great Progress in Our Pathophysiological Understanding of PA

Along with the improvement of diagnostic techniques for PA, there have been remarkable advances in pathophysiological understanding of PA. One is the development of the specific CYP11B2 antibody, and the other is the findings of somatic mutations in APAs.

The corner stone of the specific monoclonal antibody against CYP11B2 was reported in 2014 [45,46]. CYP11B2 is the rate-limiting enzyme for aldosterone synthesis and the antibody was recently used for standardization for the nomenclature and criteria of the pathological diagnosis of surgical specimens of PA patients (HISTALDO) [42]. Classically, the diagnosis of aldosterone-producing adenoma and nodule was made morphologically by HE staining of the resected specimen (classic form). On the other hand, CYP11B2 staining has made it possible to diagnose multiple aldosterone-producing nodules or, less frequently, aldosterone-producing diffuse hyperplasia. In addition, this pathological diagnosis was reproducible among different pathologists (non-classic form). This standardization of the pathological diagnosis was built up by the incorporation of a functional aspect using CYP11B2 into the diagnosis, in parallel with the conventional morphological criteria. A prospective cohort study using these diagnostic concepts showed that non-classical multiple nodules were associated with more postoperative biochemical failures compared to patients diagnosed with a single macro lesion in its classic form represented by APA (33.3 vs. 2.4 (%)) [47]. The possibility that lesions with multiple nodules may be more prone to bilateral disease should be considered, although this is a matter of speculation due to the lack of pathological findings in the residual adrenal gland. Efforts should be made to make the preoperative diagnosis closer to the pathological diagnosis in order to take advantage of the finding that the two forms of classification would have different surgical implications for PA patients. The recently published segment selective AVS (sAVS) allows us to plot the distribution of the aldosterone secretory response within the adrenal gland [34]. In particular, when partial resection was performed for the cases that showed the local distribution of aldosterone in the adrenal gland coincided with the tumor, biochemical remission was achieved in all postoperative patients, and 97% of the cases showed the pathological diagnosis of APA. In the future, these improvements in AVS diagnostic techniques, coupled with standardization of pathology diagnosis, will provide useful insights to build appropriate treatment strategies for individual patients. To this end, prospective cohort studies with uniform diagnostic criteria, pathological evaluation, and posttreatment assessment are needed to establish the frequency of APA and multiple nodules, as well as appropriate diagnostic procedures.

In parallel with these findings, the discovery of the aldosterone driver mutation has greatly advanced our understanding of the pathogenesis of PA patients. The first step in this sequence was the memorable report published in 2011, describing a somatic mutation in the *KCNJ5* gene encoding G protein-activated inward rectifier potassium channel 4 (GIRK-4) [48]. *KCNJ5* mutation was identified in 36% of APA [48,49,50]. Following this discovery, somatic mutations in *ATP2B3*, *ATP1A1* [51], and *CACNA1D* [52] were identified in 2013; although the frequency of *KCNJ5* mutations in APA is higher in Eastern countries (59.5–76.8%) [53,54,55,56] than in Western countries (34–45%) [49,50,57,58,59], *KCNJ5* mutations are similarly the most common mutation across the world. All of these mutations increase intracellular calcium concentrations, followed by activating CYP11B2 enzyme expression and increasing excess aldosterone biosynthesis. However, whether these mutations enhance cell proliferation has not been determined. Particularly, the proliferative effect seen in *KCNJ5* was different due to *KCNJ5* expression levels in the study using HAC15 [60], while another report using APAs did not show any evidence of proliferative effect of *KCNJ5* mutation [61]. However, the evidence from germline *KCNJ5* mutations suggested that *KCNJ5* mutations promote proliferation to form hyperplasia *in vivo* [62,63]. A recent study using CYP11B2 immunohistochemistry-guided high-throughput sequencing rather than Sanger sequencing identified 95% of APAs as showing one of these somatic mutations, and more APAs with *CACNA1D* mutations among *KCNJ5* wild-type APAs [64]. These mutations would play a crucial role in the pathogenesis of PA.

## 4. Pathological Insights into PA

CYP11B1 and CYCP11B2 play pivotal roles in the synthesis of adrenal corticosteroids, the former responsible for the synthesis of cortisol and corticosterone, and the latter for the synthesis of aldosterone [65]. The advent of CYP11B2 novel monoclonal antibodies has clarified the zonation of these synthesis enzymes in the normal adrenal gland [45,46]. Classically, the adrenal cortex is composed of three main zones, i.e., from the outermost layer, zona glomerulosa (ZG), to the zona fasciculata (ZF) and zona reticularis. Aldosterone and cortisol are produced by ZG and ZF cells, which have been shown by HE staining to be morphologically distinct: ZG cells are smaller, more compact, and have a smaller cytoplasm/nucleus ratio; ZF cells have a larger cytoplasm/nucleus ratio, with more fatty material and transparent appearance. Evaluation of the distribution of each synthase in the normal adrenal gland using specific CYP11B2 and B1 antibodies has shown that the distribution is different in young and elderly individuals [66,67,68]. In younger individuals, CYP11B2-positive cells are present throughout the ZG [67]; CYP11B1 was detected in both the ZF and zona reticularis. An unstained region is identified between the CYP11B2- and CYP11B1-positive cells that is not detected by either antibody. It is not clear whether this region comprises undifferentiated progenitor-like cells [69]. On the other hand, in the elderly, CYP11B2-positive cells are adjacent to the membrane and form a pattern of CYP11B2-immunopositive cell clusters with no CYP11B2 staining around them. Approximately half of the adrenals of normotensive individuals contain these regions, which have been designated as Aldosterone Producing Cell Clusters (APCCs) [66,67,68,70,71]. The nomenclature of APCC was changed to Aldosterone Producing Micronodules (APM) in the HISTALDO study [42]. The question of whether APMs have an autonomous aldosterone secretory capacity has been arisen. This is linked to the fact that the incidence of APMs correlates with the frequency of aldosterone-related hypertension [68,72]. Subsequent studies have shown that these APMs when found in adult human adrenal tissue with normal adrenal function also contain somatic mutations of the aldosterne driver mutation; mutations were identified in over 30% of APMs, most frequently in the *CACNA1D* mutation [64,66,71]. This was followed by *ATP2B3*, *ATP1A1*, and few *KCNJ5* mutations. Interestingly, in case reports suggesting APM to APA transition, APM and micro APA were adjacent, with *ATP1A1* and *ATP2B3* mutations from the former and *KCNJ5* mutations from the latter [73,74]. The research efforts have also extended to IHA cases. The study showed somatic mutations in *CACNA1D* are the cause of most IHA [75]. This is consistent with previous reports of image-negative PAs [76]. Interpretation is limited due to the small sample of surgically resected IHAs. However, their findings were much appreciated as supporting the concept of a continuum of pathophysiology from normotensive to hypertensive patients [77]. The different distribution of somatic mutations, with *KCNJ5* mutations found specifically in APA and *CACNA1D* mutations in IHA, may contribute to the difference in clinical features between APA and IHA [41].

**Figure 2:**
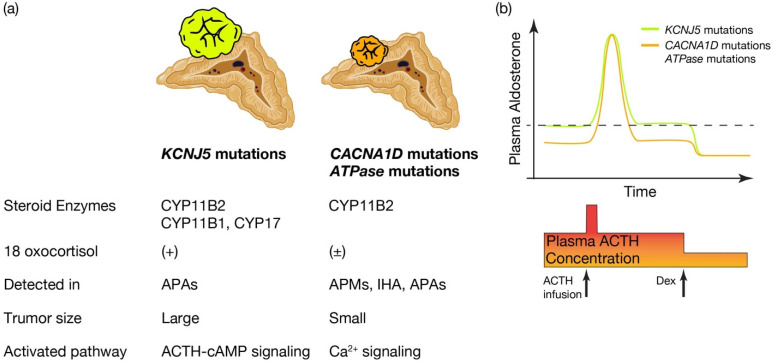
Distinctive characteristics of APA patients harboring *KCNJ5* mutations, and *CACNA1D* or *ATPase* mutations. Distinctive characteristics of aldosterone producing adenoma (APA) patients harboring *KCNJ5* mutation and *CACNA1D* or *ATPase* mutations. (**a**) *KCNJ5* mutated APAs showed larger tumor and heterogenous composition of CYP11B2, CYP11B1, and/or CYP17 positive cells, while *CACNA1D* and *ATPase* mutated APAs showed smaller tumor and homogeneous composition of CYP11B2 positive cells. 18 oxocortisol is elevated in *KCNJ5* mutated APAs, while not in *CACNA1D* and *ATPase* mutated APAs. *KCNJ5* mutations have been detected mostly in APAs, while *CACNA1D* and *ATPase* mutations have been dominantly identified in aldosterone producing micronodules (APMs) and idiopathic hyperaldosteronism (IHA) as well as APAs. We assume distinctive activated pathways of aldosterone synthesis between *KCNJ5* mutated and *CACNA1D* or *ATPase* mutated APAs, such as the ACTH-cAMP and Ca^2+^ signaling pathways. (**b**) Conceptual scheme of responsiveness of aldosterone secretion to ACTH between APAs harboring *KCNJ5* and *CACNA1D* or *ATPase* mutations is shown. Basal aldosterone secretion is higher and its responsiveness to ACTH stimulation is lower in *KCNJ5* mutated APAs than *CACNA1D* and *ATPase* mutated APAs. ACTH depletion via dexamethasone (Dex) suppression decreased plasma aldosterone levels from *KCNJ5* mutated APAs to that from *KCNJ5* wild APAs [55,81,82]. Thus we assume ACTH-cAMP signaling in KCNJ5 mutated APAs is activated to increase basal aldosterone secretion and to lessen response to extra ACTH stimulation. Figure was created with BioRender.com.

What is the pathological characteristics of APA harboring *KCNJ5*, and are they defined by the expression profile of steroidogenic enzymes? (See Figure 2a.) *KCNJ5* mutated APAs are larger than other APAs; 60% are composed of lipid-laden clear ZF like cells and 40% are composed of compact ZG like cells [78]. Immunohistological studies on steroid synthase have shown that *KCNJ5* mutated APAs are positive for CYP11B2 alone, co-expressing CYP11B2 and CYP17, and in a few cases, cells co-expressing CYP11B2, CYP11B1 and CYP17 [46,78,79]. On the other hand, APA with ATPA2B3 mutations tended to be dominated by compact ZG like cells, while APA with *ATP1A1* and *CACNA1D* mutations showed heterogeneity from tumor to tumor with no clear advantage [55,78]. Very interestingly, the expression of CYP11B2 and CYP17A1, which are involved in aldosterone and cortisol synthesis, correlated positively in *KCNJ5* mutant APAs, but negatively in *ATP2B3* mutants [78]. The correlation between the two is not clear for *ATP1A1* or *CACNA1D*. Inconsistent with this profile of steroidogenic enzymes, the normally negligible hybrid steroid, 18 oxocortisol (see the section “*Development of prediction model for somatic KCNJ5 mutation in PA patinets*”), is markedly increased in patients with APAs harboring *KCNJ5* mutations. This increase has not been observed in patients with APAs harboring *ATPase* and *CACNA1D* mutations [64,80] (Figure 2a). This series of results has suggested that *KCNJ5* mutations are associated with the formation of APA and that the pathogenetic process differs from that of *ATPase* and *CACNA1D* mutations. APAs harboring *KCNJ5* mutations may have a more disorganized process of tumor cell differentiation and formation than APAs with *ATPase* or *CACNA1D* mutations. It has not been clear how the *KCNJ5* mutation occurred in APAs. Considering the high prevalence of the *KCNJ5* mutation in Asian populations, variant SNPs specific to Asian races or epigenetic changes might be derived from environmental situations, such as high salt intake, which might affect mutation frequency. An aldosterone synthesis pathway of the latter deviates from the zonation-based steroid synthesis. This may be reflected in the specific clinical features.

## 5. Clinical Implication of *KCNJ5* Somatic Mutation in APA

**Figure 3:**
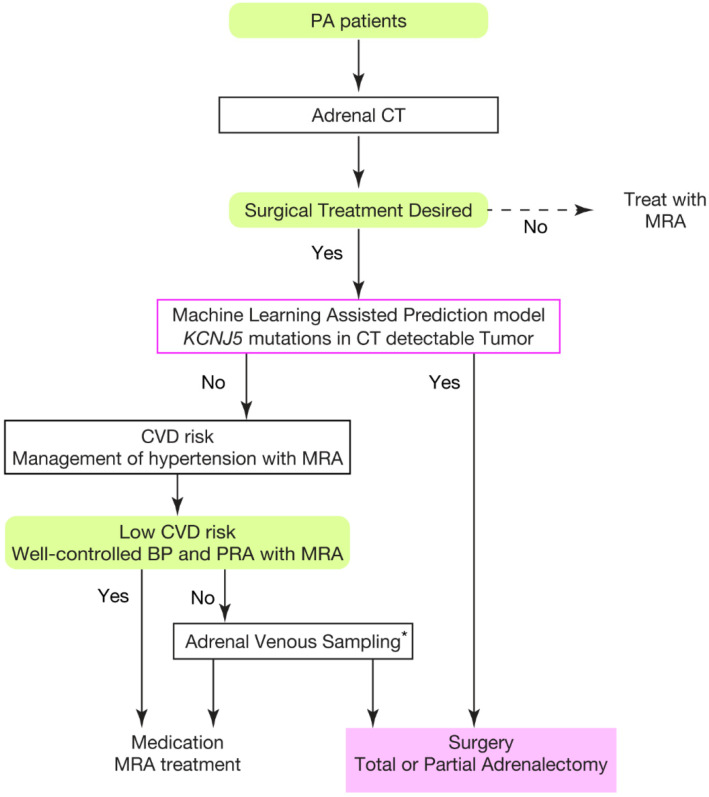
Conceptual scheme of treatment strategy based on prediction model for *KCNJ5* mutations in CT detectable tumor. Conceptual scheme of utilizing presurgical diagnosis of *KCNJ5* mutations in CT detectable tumors. CVD risk indicates (1) current cardiovascular complications and (2) future risk of CVD events. To achieve well controlled blood pressure (BP) and PRA, BP should be controlled to the same level as essential hypertensives, and PRA should be elevated more than 1.0 ng/mL/h with MRA. * Adrenal venous sampling should be performed for the cases with *KCNJ5*-wild APAs who can receive surgical benefits, such as MRA-resistant primary aldosteronism of uncontrollable blood pressure and plasma renin. Debulking of APA for bilateral APA cases and adrenalectomy for unilateral APA cases should alleviate the hormonal abnormality in this population. Otherwise, the prognosis should be similar with appropriate treatment with MRA to essential hypertensive patients due to a lower outcome of complete clinical success in *KCNJ5* wild-type APA cases than in *KCNJ5* mutated APA cases. PA, primary aldosteronism; CT, computed tomography; CVD, cardiovascular disease; BP, blood pressure; PRA, plasma renin activity.

The intriguing question regarding the discovery of the *KCNJ5* mutation is whether it could change our treatment strategy. To begin with, when a definite diagnosis of APA is made, more than 95% of these patients are in biological remission [40]. Thus, resection of APA with or without mutation has been shown to improve patient prognosis. However, the clinical prognosis of patients with resection of unilateral lesions has been shown to vary widely, with 33–77% of hypertensive cases in remission [83,84,85,86]. Therefore, research is underway to determine which patients will achieve remission. Common predictors of hypertensive remission after surgery for unilateral PA patients [83,84,85,86] have included gender, body mass index (BMI), duration of hypertension, and number of antihypertensive drugs. When PA patients are treated appropriately with MRA, i.e., to a PRA > 1.0, their risk can be improved to that of patients with essential hypertension [38]. Thus, when PA is biochemically in remission after surgery without remission of hypertension, most patients are considered to have essential hypertension; the risk of cardiovascular events may only be reduced to the same level as that of PA cases whose plasma renin suppression is appropriately treated. Therefore, surgery should be performed as early as possible in patients who are difficult to manage with MRA, as well as in those who are supposed to be in remission (Figure 3). Clinical features of patients with *KCNJ5* mutated APA have included younger age, higher plasma aldosterone levels, lower potassium, and having larger tumors than those with *KCNJ5* wild APAs [48,49,50,57,59,87,88] (Figure 2a). In a retrospective cohort study scrutinizing more than 100 patients, cardiac hypertrophy, frequently seen in PA cases, was shown to improve significantly in those who harbored *KCNJ5* mutated APAs after unilateral adrenalectomy compared to those with *KCNJ5* wild APAs [89,90]. In addition, the *KCNJ5* mutation was found as an independent remission factor. A subsequent large prospective cohort study using propensity score matching showed that surgery in the cases with APA harboring *KCNJ5* mutation group significantly improved left ventricular hypertrophy compared to the *KCNJ5* wild group [91]. Interestingly, in this study, when the outcome was left ventricular diastolic function, measured through a functional assessment of left ventricular hypertrophy, the benefit of adrenalectomy was seen only in the *KCNJ5* mutant group and not in the *KCNJ5* wild type group. Analysis has also been conducted using a retrospective cohort to evaluate the impact of the *KCNJ5* mutation on the remission of hypertension. In each case, possession of the *KCNJ5* mutation was found to be an independent factor for remission of postoperative hypertension [90,92]. Recently, however, a prospective cohort study analyzing 45 patients was reported, in which there was no difference in hypertension remission rate according to the presence or absence of *KCNJ5* mutation [47]. This point should be tested in future prospective cohorts with sample sizes that allow propensity score matching for age, gender, body mass index, blood pressure, duration of hypertension, and number of hypertension medications. Long-term data that clarify the incidence of cardiovascular complications and risk of mortality are particularly desirable.

## 6. Development of Prediction Model for Somatic *KCNJ5* Mutation in PA Patinets

While the clinical importance of *KCNJ5* mutations is being revealed, the findings of aldosterone driver mutations have been still far from clinical application, as they can currently be diagnosed only with surgical specimens. Is it possible to predict *KCNJ5* mutations preoperatively? Prediction strategies should include: (1) direct detection of tissue DNA of *KCNJ5* mutant APAs that may be leaked into the blood (a.k.a., liquid biopsy), (2) search for new biomarkers characteristic of *KCNJ5* mutant APAs, and (3) pattern recognition using known clinical features (e.g., machine learning method). Unfortunately, there have been no reports of successful liquid biopsy to detect *KCNJ5* mutation in APA to date. As an example of a new biomarker discovery, steroid profiling has shown very promising potential for preoperative diagnosis [80,93,94,95]. Cortisol is synthesized by conversion of pregnenolone and progesterone to 17-hydroxypregnenolone and 17-hydroxyprogesterone by the CYP11B1 enzyme expressed in ZF. The 11-Deoxycortisol and cortisol produced by this pathway are then synthesized into the hybrid steroid 18-Oxocortisol and 18-hydroxycortisol by CYP11B2, which is normally expressed in ZG. These hybrid steroids are detected only in trace amounts in normal adrenal glands, where zonation is clearly distinguished. However, their blood levels are elevated in APA, especially in the presence of *KCNJ5* mutations [80,93]. As mentioned earlier, in APA with *KCNJ5* mutation, the expression of CYP11B2 and CYP17A1 is positively correlated, and although rare, cells co-expressing CYP11B1 have also been identified. The results may support these pathological features. Although there was wide variation, APAs with *KCNJ5* mutations had up to 18-fold higher levels of aldosterone, 18-oxocortisol, 18-hydroxycortisol, and 11-deoxycorticosterone in the adrenal veins compared to APAs of the other genotypes [93]. In peripheral blood, 18-oxocortisol was elevated up to 18-fold. Using these characteristic results, the predictive model that was built allowed us to fractionate each mutation with 95% accuracy using adrenal venous blood, with *KCNJ5* and *CACNA1D* being 100% accurate. Furthermore, combining this mass spectrometry-based plasma steroid profiling with machine learning enabled the diagnosis of the presence of primary aldosteronism and APA with *KCNJ5* mutations with sensitivity of 69% and 85%, and specificity of 85% and 97%, respectively [95]. Steroid profiling may be useful for screening PA and detecting the cases with APA, especially harboring *KCNJ5* mutations.

The development of mutant *KCNJ5* channel blockers also has potential diagnostic tools as well as therapeutic utility. A reduction in aldosterone secretory responsiveness to therapy may predict *KCNJ5* mutations when obtained. Candidate drugs include calcium antagonists and macrolide compounds that specifically inhibit mutant *KCNJ5* channels and blockers of Na^+^-Ca^++^ transport proteins, such as the calcium blockers verapamil and amiloride [96,97,98,99,100]. Drug screening has also confirmed that macrolide compounds reduced aldosterone secretion from *KCNJ5* mutant APA cells *in vitro* and *ex vivo*. However, results regarding the diagnostic application of PA in patients are pending [101].

Finally, as for establishing a prediction method based on pattern recognition using a clinical values commonly used in the PA diagnostic steps, although papers using machine learning for APA are beginning to be seen [30,102,103], there have been currently no significant results on the detection of *KCNJ5*-mutated APAs. However, several other clinical characteristics of APA patients with *KCNJ5* mutations have been reported: younger age, female dominant, lower potassium level, higher aldosterone value, and large tumor size compared to *KCNJ5* wild APA cases [48,49,50,57,58,59,87,88]. Compared to cases with APA harboring *ATPase* or *CACNA1D* mutation, *KCNJ5* mutated APA cases showed less responsiveness to ACTH stimulation with higher basal value of plasma aldosterone [81]. This finding is consistent with the result of ex vivo study [55]. Conversely, when ACTH is suppressed by Dexamethazone, aldosterone secretions are found to be more suppressed in *KCNJ5* mutated APA cases than in *KCNJ5* wild type cases, resulting in no statistical difference in plasma aldosterone value between them [82]. These results suggest that APAs with *KCNJ5* mutations have an increased sensitivity to ACTH signaling, which is constantly stimulated and raises basal aldosterone secretion (Figure 2b). On the other hand, responsiveness to the ATII system is not clear. In addition to basic clinical data, the combination of tests that manipulate the ATII system, such as the saline infusion or captopril challenge tests, and tests that stimulate ACTH signaling, such as the ACTH infusion or Dexamethazone suppression tests, may improve the predictive power of *KCNJ5* mutations.

If these findings allow us presurgical diagnosis of *KCNJ5* mutations in APA by pattern recognition of machine learning using new biomarkers or widely available clinical information, the treatment strategy for PA will change dramatically. Since *KCNJ5* mutations are often identified in APA and we need to recognize APA location for surgical treatment, it is important to determine whether CT-recognizable tumors carry *KCNJ5* mutations. If a visible tumor is predicted to have *KCNJ5* mutation, adrenalectomy is recommended without AVS for the prevention of cardiovascular complications and better prognosis. If the tumor does not carry the *KCNJ5* mutation, AVS should be recommended to those who receive surgical benefit. The surgical indication should be considered based on the patient’s current cardiovascular complications, risk of future cardiovascular disease, and response to MRA to manage blood pressure of PA. Otherwise, we can manage PA cases with MRA without further testing (Figure 3).

## 7. Conclusions

Elucidation of detailed pathophysiology and appropriate classification of PA are the fundamental to achieve individualized medicine for PA patients. In the past decade, the development of specific CYP11B2 antibodies and the discovery of the aldosterone driver mutations have largely progressed our understanding of primary aldosteronism. The pathological significance of *KCNJ5* somatic mutation is also of great importance for understanding the origin of APA: the most frequent aldosterone driver mutation in APA is very rare in AMN, while *CACNA1D* is most common in AMN and diffuse hyperplasia. These results suggest the possibility that they may have different pathogenesis. A further question to be answered clinically is whether APAs with *KCNJ5* mutations lead to more severe cardiovascular complications and shorter healthy life expectancy compared to those with *KCNJ5* wild type. The answer would determine if it is worthwhile making predictions before surgery to provide individualized treatment. The studies obtained to date have indicated a more effective surgical approach for *KCNJ5* mutated APA patients, but the long-term therapeutic benefit is not clear. Prospective cohort or randomized control studies are expected to provide evidence for targeted treatment of APAs harboring *KCNJ5* mutations.

## Abbreviations

AVS	Adrenal Venous Sampling
PA	Primary Aldosteronism
sAVS	Segmental selective AVS
IHA	Idiopathic hyperaldosteronism
cAVS	Central AVS
APA	Aldosterone-producing adenoma
